# Translational Devices, Technologies, and Medicines in Clinical Ophthalmology

**DOI:** 10.1155/2017/2876896

**Published:** 2017-01-15

**Authors:** George M. Saleh, M. Reza Vagefi, Ioannis Athanasiadis

**Affiliations:** ^1^The National Institute for Health Research Biomedical Research Centre at Moorfields Eye Hospital and UCL Institute of Ophthalmology, London, UK; ^2^Oculofacial Plastic, Orbital and Reconstructive Surgery, University of California, San Francisco, San Francisco, CA, USA; ^3^Modern Ophthalmic Practice, Thessaloniki, Greece

Over the last few decades, there has been a rapid advance in the technologies contributing to clinical ophthalmology ranging from novel hardware and software to new imaging and laser modalities with the emergence of nanotechnologies and drug innovations. The stated aim of translational research is to apply discoveries from basic science to enhance medical practice and human health, with many of these already having a major impact. We are presently witnessing an array of developments emerging on the horizon that have the potential to enhance patient care, improve diagnosis, and deliver treatments.

Wearable sensors for health and activity monitoring are growing in popularity with a multitude of devices, such as Fitbit and Withings, coming to market able to monitor vitals and measure activity levels and calorific consumption and expenditure. Their scope is now being extended to monitor disease as well. The application of contact lens sensors in ocular diagnostics offers a minimally invasive platform for constant monitoring of pertinent disease indices [1]. Advances in materials, electronics, and microfabrication techniques have expanded the remit of this medical device from a visual corrective aid in isolation to one that has diagnostic potential. The possibilities of its application have even caught the interests of Google, Novartis, and Microsoft [2–4]. Within this issue, S. C. Xu and colleagues review the applications of contact lens sensors (CLS) in detecting 24-hour intraocular pressure with its potential to change our approach and understanding in glaucoma. CLS have also been used to quantify blink rate and limbal strain and measure the circadian rhythm in a variety of disease states including normal tension glaucoma and thyroid eye disease.

The ever-increasing power of computational hardware (as described by Moore's Law) coupled with accelerating advances in software engineering has allowed swift evolution of this technology in ophthalmology. This special issue provides a report on an automated diabetic retinopathy detection software system using singular spectrum analysis to focus on microaneurysms. When applied to over 17,000 retinal images across different racial groups from six different countries, the authors report high levels of performance and the potential for scalability in diverse populations. Computational power also underpins virtual reality modeling and simulation has been used with success in nonmedical domains such as aerospace and the military. Inroads with this technology are now being made in medicine with cancer and surgical applications [5–7]. In this issue, E. Lanchares et al. describe a finite element model of the eye to analyze the myopic effects of scleral buckling, concluding that the wider the band, the greater the induced myopia. This and other ophthalmic models will likely grow in power as further clinical correlation is sought, a greater understanding of ocular biomechanics is ascertained, and improved boundary conditions are applied.

Optical coherence tomography angiography (OCTA) involves noninvasive imaging that rapidly generates high-resolution volumetric angiography and visualization of blood flow without injection of a contrast agent. As a result, an indirect representation of the vascular morphology of the retina and choroid is generated. This nascent technology has wide potential applicability for retinal vascular disease; however, OCTA requires higher imaging speeds than what most currently available OCT systems can provide in order to obtain a densely sampled volume. Its current limitations also include a relatively small field of view, inability to show leakage, and proclivity for image artifact due to patient movement. Nevertheless, with recent developments in equipment and software, OCTA can now measure the size of choroidal neovascularization (CNV). Q. Chen et al. assess the morphology of idiopathic choroidal neovascularization (ICNV) by OCTA in 17 patients undergoing intravitreal antivascular endothelial growth factor treatment. Future work will continue to define the applications of this contrast-free angiography to retinal disease.

Crowdsourcing is a relatively novel, cost-effective endeavour involving the distribution of work (“outsourcing”) to a large group of people (the “crowd”) typically via the Internet where the collective intelligence and combined efforts of networked communities are used for specific purposes such as data analysis. Crowdsourcing is increasingly used commercially and more recently for scientific research such as the Zooniverse project [8, 9]. Due to the strict regulation governing medical research, crowdsourcing has been slow to be adopted but still has significant potential in medicine and ophthalmology [10]. In this issue, A. Y. Lee et al. evaluate the feasibility of using Mechanical Turk to perform manual segmentation of macular OCT images. The Amazon Mechanical Turk, which derives its name from the fake chess-playing automaton of the 18th century, is one of the most popular crowdsourcing Internet marketplaces for the coordinated use of human intelligence tasks (HITs) that computers are currently unable to do. This novel proof-of-concept study concludes that Mechanical Turk provides a cost-effective, scalable, high-availability infrastructure for manual segmentation of OCT images. As large data sets are becoming increasingly common with today's clinical studies and multicenter trials, rapid, reliable, cost-effective methods of interpretation will be crucial.

In this special issue on translational devices, technologies, and medicines in clinical ophthalmology, various potential breakthrough developments are reported in diverse spheres of ophthalmic practice. It is an exciting time for ophthalmology as we sit on the precipice of many significant advances that will steer the progress of our discipline. These innovations will inevitably improve the detection of disease and provide cutting-edge medical therapeutics and surgical interventions for our patients.

*George M. Saleh* *George M. Saleh* *M. Reza Vagefi* *M. Reza Vagefi* *Ioannis Athanasiadis* *Ioannis Athanasiadis*
